# Cellulose Nanocrystal and Self-Assembling Lignin Enhanced the PEDOT/PSS/PVA Composite on Mechanical and Self-Powered Wearable Properties

**DOI:** 10.1021/acsomega.4c07933

**Published:** 2025-04-10

**Authors:** Shih-Chen Shi, Yan-Ching Hsieh, Dieter Rahmadiawan

**Affiliations:** † Department of Mechanical Engineering, National Cheng Kung University, Tainan 70101, Taiwan; ‡ Department of Mechanical Engineering, Universitas Negeri Padang, Padang, Sumatera Barat 25173, Indonesia

## Abstract

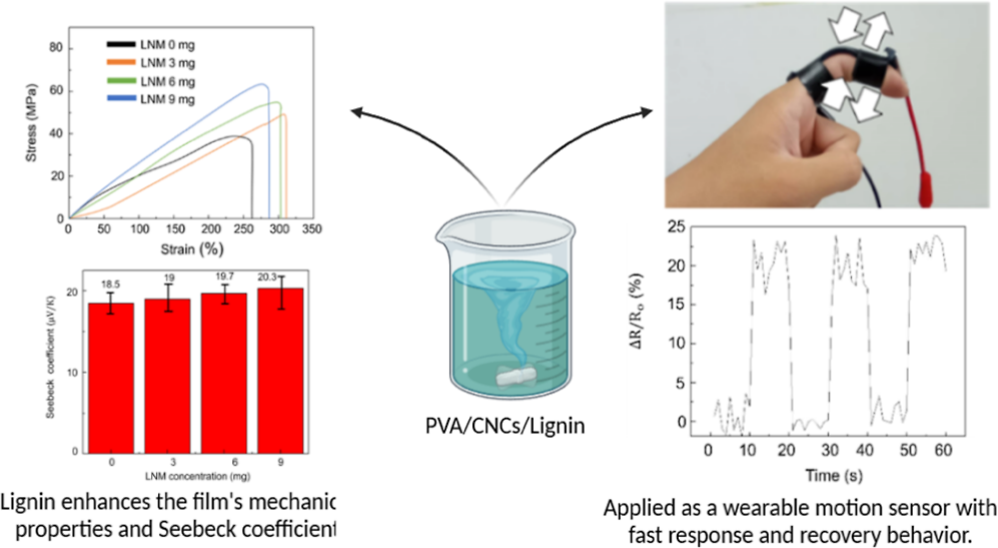

Lignin nanomicelle (LNM) synthesis via deep eutectic solvent (DES) has been optimized from a conventional duration of 2–3 days to a streamlined 12 h procedure utilizing autoclave reactor heating. This approach facilitates the efficient extraction of lignin from straw and its subsequent formation into LNMs via a simultaneous self-assembly mechanism. Integration of these amphiphilic LNMs into a cellulose nanocrystal (CNC) framework, combined with PEDOT: PSS in a poly(vinyl alcohol) (PVA) matrix, yields a self-powered strain sensor characterized by enhanced tensile properties and heightened strain sensitivity. Incorporating carboxyl functional groups from LNMs on the PVA matrix significantly augments the sensor’s mechanical strength and elasticity. This is evidenced by achieving Young’s modulus of 65.9 MPa and an elongation capacity of 320%, ensuring its efficacy in human motion detection. The synergistic inclusion of CNCs and LNMs amplifies the sensor’s gauge factor, thereby augmenting its strain responsiveness. The elevated aspect ratio of CNCs establishes an efficacious electrical network that, in concert with the interaction between CNCs and PEDOT: PSS, diminishes the electrical percolation threshold, culminating in an improved gauge factor of 19, indicative of enhanced strain detection capabilities. Furthermore, the sensor can generate a thermoelectric voltage in response to thermal gradients, with the dynamic structures of LNM improving the conductivity and PEDOT: PSS dispersion within the PVA matrix, thereby optimizing the Seebeck coefficient. After enduring 5000 cycles of 100% strain deformation tests, the sensor demonstrates consistent performance, underscoring its reliability and durability. The fabricated PVA/Gly–LNM/CNCs/PEDOT: PSS composite material has been successfully applied to detect nuanced human gestures, including finger and wrist movements, affirming its potential utility in wearable technology applications.

## 1 Highlights

1. Autoclave heating reduces lignin nanomicelle synthesis to 12 h, significantly streamlining production.
2. Integrating amphiphilic lignin nanomicelles with a cellulose nanocrystal network yields a self-powered strain sensor with exceptional sensitivity.
3. Achieving Young’s modulus of 65.9 MPa and 320% stretchability, the sensor is adept at precisely detecting human movements.
4. The sensor’s sensitivity, indicated by a gauge factor of 19, is markedly enhanced through the synergy of cellulose nanocrystals and lignin nanomicelles.
5. Proven durable over 5000 cycles, the sensor showcases its capability for wearable technologies, specifically in nuanced motion detection.

## 2 Introduction

Wearable devices encompass mobile electronic gadgets affixed directly to the human skin or woven into clothing and accessories. These devices have revolutionized how we monitor health, offering capabilities to track vital signs, sleep patterns, and neurological states, and even function as specialized monitors like electrocardiographs and fetal and obstetric care devices. Beyond health monitoring, wearable technology extends to smartwatches and intelligent apparel, unlocking new possibilities for tracking and understanding physiological and biological conditions over time.^1−5^

The push toward developing high-performance, multifunctional, flexible electronics has significantly advanced our capacity for seamless human-machine interaction, clinical diagnostics, and therapeutic interventions. Innovations in skin-like electronics facilitate real-time monitoring of physical activities, setting the stage for the next wave of sensors that prioritize elasticity, minimal electrical resistance, and hydration maintenance to ensure accurate signal capture.^6−8^

Multifunctional conductive hydrogel sensors represent a promising area of development due to their superior mechanical flexibility, electrical conductivity, and biocompatibility, making them highly suitable for wearable and biomedical applications.^9^ Conductive hydrogels, which can simultaneously sense temperature, strain, and chemical changes, provide unique advantages in wearable sensors by leveraging their self-healing capabilities, stretchability, and high sensitivity to environmental stimuli.^10^

The rising interest in wearable and electronic skin technologies can be attributed to their adaptability, affordability, and environmental efficiency. The exploration of elastic polymer composites, hydrogels, and other materials aims to satisfy the increasing demand for responsive strain sensors. Promising developments in flexible nanocomposites, utilizing bases like natural rubber, polyethylenimine, poly(vinyl alcohol), polydimethylsiloxane (PDMS), and polyurethane (PU), highlight their robust mechanical properties and longevity.^12^ Adjusting these composites’ electrical conductivity is simplified by including conductive fillers—nanometal particles, carbon nanotubes, graphene, and conductive polymers—though attention must be paid to how these additives influence the materials’ elasticity and responsiveness.^13−16^

Despite these advancements, the reliance on external power sources poses challenges, especially in conditions where sustainability is paramount. This underscores the importance of designing wearable sensors that are not only highly sensitive and flexible but also self-sufficient, ensuring their effectiveness in diverse environments and contributing to the broader goal of sustainable technological development.

PEDOT: PSS is one of the most favored organic thermoelectric materials today, prized for its nontoxic, flexible, and straightforward manufacturing advantages.^17,18^ Cellulose nanocrystals (CNCs) are single-carbon nanowires known for their distinctive helical structures.^19,20^ These CNCs can bind conductive materials via their surface’s rich functional groups, such as hydroxyl, sulfate, and carboxyl.^21,22^ The amphiphilic nature of CNCs notably enhances the dispersion of various conductive materials, including graphene and conductive polymers, due to point-to-point contacts among CNCs, showcasing their superior strain-sensing capabilities.^23−26^ Hydrophilic lignin nanomicelles (LNMs), prepared using deep eutectic solvents (DESs), represent a green and manageable processing method.^12,27−29^ DES is highly effective at dissolving and valorizing lignin, forming spherical hydrophilic surfaces that facilitate the even distribution of LNMs within a poly(vinyl alcohol) (PVA) matrix.^30^ Incorporating glycerol into hydrogels is a viable strategy to boost their antifreeze and moisturizing attributes.^31,32^ It has been observed that increasing glycerol (Gly) content tends to lower the composite material’s Young’s modulus.^33^ In contrast, the enhanced ductility in PVA/Gly-CNCs/polyvinylpyrrolidone/poly(3,4-ethylenedioxythiophene) (PEDOT) composites stems from glycerol’s plasticizing effect, increasing the PVA chains’ mobility.^16,34,35^

In this study, glycerol-enriched PVA is the foundation for an elastic polymer composite, with LNM included to bolster the conductive polymer PEDOT: poly(styrenesulfonate) (PSS). By integrating CNCs, a structural framework is constructed within the conductive polymer hydrogel, ensuring PEDOT: PSS molecules are evenly dispersed in PVA, culminating in the creation of a self-powered, wearable strain sensor PVA/Gly–CNCs/PEDOT: PSS/LNM. PEDOT: PSS, acting as the organic thermoelectric component alongside the flexible PVA substrate and CNCs as nanoadditives, helps lower the permeation threshold and improve the conductive materials’ dispersion within the composite matrix. As the ideal plasticizer for PVA composites, glycerol enhances elasticity and decreases Young’s modulus. The inclusion of self-assembled LNMs, with its amphiphilic properties, further boost the dispersion and conductivity of various conductive materials. The composite PVA/Gly–CNCs/LNM/PEDOT: PSS leverages the Seebeck effect for its self-powering functionality.

## 3 Materials and Experiment

### Lignin Nanomicelle Preparation

LNMs were synthesized through a hydrothermal process that extracts lignin from straw using DESs, simultaneously prompting the lignin to self-assemble into LNMs. The choline chloride–lactic acid-based DES mixture is prepared with a molar ratio of 1:2 between choline chloride and lactic acid. In a vacuum oven, this mixture is melted at 60 °C for 2 h. Following this, 3g of dewaxed straw is introduced into the explicit molten DES solvent, and the DES-soaked straw is subjected to a hydrothermal reaction in an autoclave.

After the reaction, the straw residue was filtered, and the DES-lignin solution was collected. Lignin was precipitated by diluting the DES-lignin solution with an equal volume of deionized water, followed by acidification with 2 M HCl to adjust the pH to ∼2. The precipitated lignin was separated by centrifugation at 10,000 rpm for 10 min, washed thoroughly with DI water to remove residual DES, and dried under vacuum at 40 °C. During this process, the lignin self-assembled into nanomicelles due to hydrophobic interactions.

The chemical composition of LNMs was analyzed using the method developed by the National Renewable Energy Laboratory (NREL).^36^ The purity of LNMs is calculated based on the sum of acid-insoluble lignin and acid-soluble lignin. Particle size analysis of LNMs is conducted using field emission scanning electron microscopes, with the assistance of ImageJ, to differentiate particles from the matrix and then segment the unfilled matrix areas to calculate particle size.

### Self-Powered Strain Sensor Preparation

About 200 mg of PVA powder (Sigma-Aldrich, 1799) is added to 200 mL of DI water and heated in an oven at 90 °C for 2 h. For samples with glycerol, the appropriate amount (as specified by the sample name) is added and stirred magnetically for 30 min to ensure uniform distribution. Once the PVA is completely dissolved in the solution, the PVA solution is cooled to room temperature. Three g of CNC powder is added to 100 mL of pure water and stirred magnetically for 2 h. LNM is added to dimethyl sulfoxide (DMSO, Sigma-Aldrich) and sonicated for 20 min. The dissolved LNM solution is then mixed with different ratios of PEDOT: PSS and sonicated for another 20 min to create LNM/PEDOT: PSS. Ten ml of the CNC solution is added to the PVA solution, followed by the addition of the LNM/PEDOT: PSS solution into the PVA/CNCs solution and magnetically stirred for 2 h. After thorough stirring, the mixture solution is poured into molds. The molds are then placed in the freezer, frozen for 2 h, and thawed for 1 h, repeating this process 3 times. Table 1 shows the complete material composition of all samples.

**Table 1:** Material Composition of all Samples

sample name	PVA (mg)	CNCs (g)	LNM (mg)	glycerol (wt %)	PEDOT (mg)	PSS (mg)	DMSO (ml)
LNM 0 mg	200	0.3	0	0	1	1	0
LNM 3 mg	200	0.3	3	0	1	1	0
LNM 6 mg	200	0.3	6	0	1	2	0
LNM 9 mg	200	0.3	9	0	1	3	0
glycerol 0 wt %	200	0.3	0	0	1	1	0
glycerol 10 wt %	200	0.3	0	10	1	1	0
glycerol 20 wt %	200	0.3	0	20	1	2	0
glycerol 30 wt %	200	0.3	0	30	1	3	0
DMSO 0 m	200	0.3	6	10	2	4	0
DMSO 3 mL	200	0.3	6	10	2	4	3
DMSO 5 mL	200	0.3	6	10	2	4	5
DMSO 10 mL	200	0.3	6	10	2	4	10

### 3.1 Physical, Mechanical, Chemical, and Thermoelectric Characteristics Analysis of Composites

FTIR analysis was performed using a Fourier transform infrared spectrometer (Nicolet iS5, ThermoFisher Scientific, USA) to identify the functional groups and chemical interactions in the composite films. The surface morphology of the composite films was examined using a scanning electron microscope (Hitachi SU5000, Japan), operating at an accelerating voltage of 10 kV to study the dispersion and integration of CNCs and LNM into the PVA matrix. Physical analysis was investigated with an XRD spectrometer (Bruker D8 Venture, Bruker, Canada) over a 2θ range from 5° to 80°. Material tensile tests were conducted using a Universal Testing Machine (Material Testing System, AGS–X, SHIMADZU, Japan), following the ASTM D882 standards for specimen preparation, with all parameters undergoing more than five tensile tests. Thermoelectric properties were assessed by cutting the prepared composite films into rectangles measuring 40 × 5 mm, connecting both ends of the composite film with a multimeter (PROVA–803) to measure the thermoelectric voltage and current. The strain-sensing performance of the strain sensor is tested on a custom-made strain rig, utilizing the dual-probe technique to measure all instantaneous responses, with resistance changes during stretching calculated using Ohm’s law (*R* = *V*/*I*). The calculation of the relative resistance change and the gauge factor (GF) are shown in eqs 1 and (2), respectively.
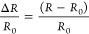
1
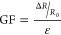
2

*R*_0_ represents the initial resistance, *R* is the real-time resistance, and ε is the applied strain.

### 3.2 Self-Powered Thermoelectric Properties, Fatigue Testing, and Human Motion Behavior Detection

A cooling chip is connected to one end of the composite film to generate a temperature difference. The temperature signal is measured by a thermometer (UNi-T UT325), and as the temperature difference (Δ*T*) increases, the voltage is measured. The Seebeck coefficient is obtained by linearly fitting the curve of Δ*T* and voltage. The self-powered strain-sensing performance of the strain sensor is tested on a custom-made strain rig. The strain sensor undergoes 10,000 cycles of stretching and compression on a custom-made cyclic stretching–compression rig, after which the sensor is tested for its strain-sensing performance on the same rig to evaluate its fatigue response to cyclic deformation. The sensor is then adhered to various human body parts, such as fingers, elbows, and wrists, to monitor real-time strain responses to human motion. A multimeter (PROVA–803) is connected to the strain sensor to measure instantaneous resistance changes, thereby capturing human movement dynamics.

## 4 Results and Discussion

### 4.1 Analysis of LNMs

**Figure 1:**
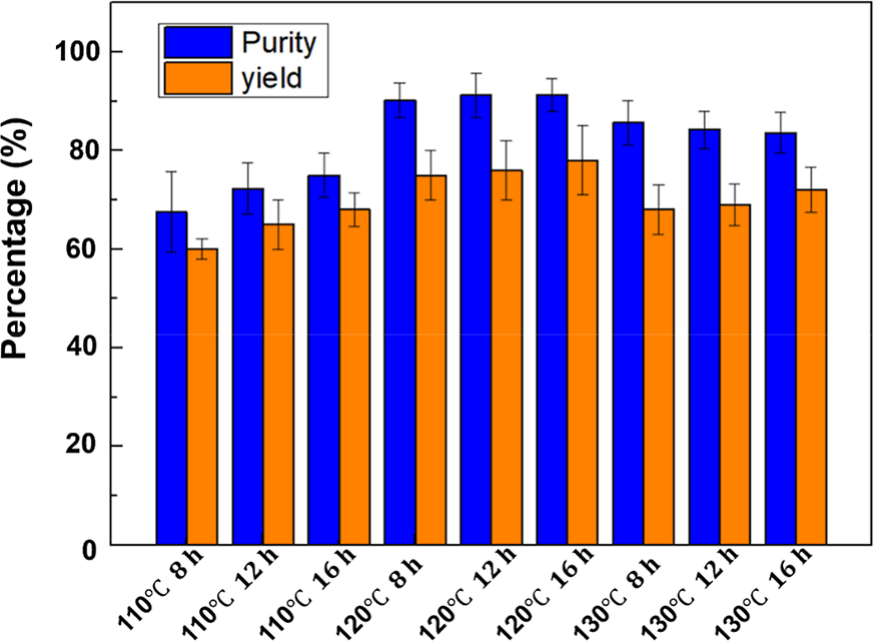
Purity and yield of LNMs produced by the DES method.

The purity and yield of LNMs extracted under different treatment conditions are shown in Figure 1. Although extraction rates vary from 60% to 78%, the purity of lignin extracted using DES consistently exceeds 60%. The purity of most LNM samples (LNM_120 °C–8h_, LNM_120 °C–12h,_ LNM_120 °C–16h_) reaches over 90%. The optimal LNM extraction results considering both yield and purity were obtained under two conditions: L_120 °C–12h_ (with a purity of 91.2% and yield of 75%) and L_120 °C–16h_ (purity 91.3%, yield 78.1%).

High-purity LNM is extracted from lignocellulosic biomass using ChCl–LA-based DES.^37,38^ The ChCl–LA DES has a high affinity for LNM, allowing for lignin extraction from straw. The purity of LNM is influenced by the reaction time and temperature of DES treatment, as shown in Figure 1. At 110 °C, LNM yield is generally lower within 16 h, but the purity is good and increases with treatment time. Increasing the treatment temperature to 120 °C significantly improves LNM yield and lignin purity. Extending the treatment time further increases lignin yield without improving purity. Processing wheat straw at a higher temperature of 130 °C does not increase the extraction rate or purity of LNMs. In the cases of LNM_130 °C–8h_, LNM_130 °C–12h_, and LNM_130 °C–16h_, a decrease in lignin purity is detected as the treatment time extends. The purity decrease at 130 °C is due to the possibility that increasing the treatment temperature may promote biomass degradation or lignin. Some lignin dissolved after 12 and 24 h of high-temperature DES treatment reprecipitates onto the residual solids of the straw, leading to a decrease in LNM purity at 130 °C.^39^

### 4.2 Self-Assembled LNM Particle Morphology and Size

**Figure 2:**
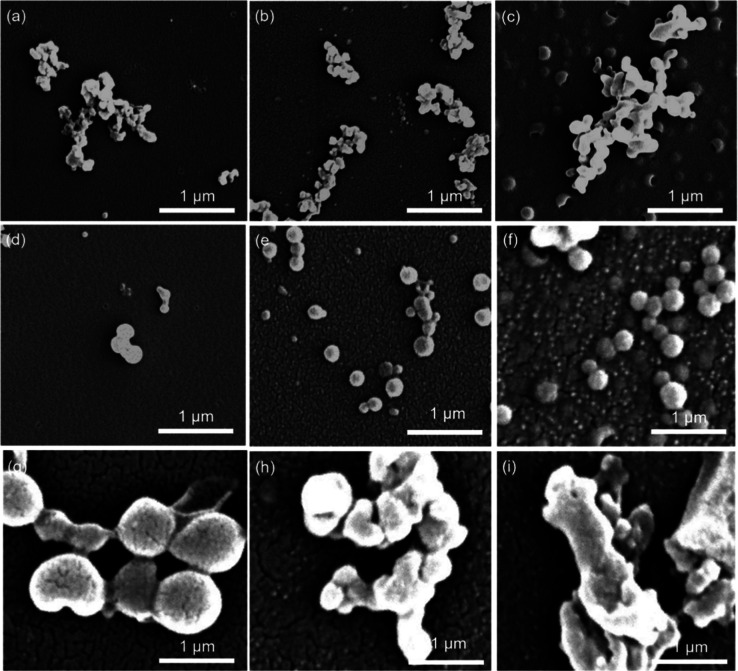
Surface morphology of LNMs at 110 °C 8 h (a), 110 °C 12 h (b), 110 °C 16 h (c), 120 °C 8 h (d), 120 °C 12 h (e), 120 °C 16 h (f), 130 °C 8 h (g), 130 °C 12 h (h), and 130 °C 16 h (i).

DES treatment mainly produces dispersed lignin particles within the nanoscale, as shown in Figure 2. Besides the cleavage of ester and ether bonds, more vital DES treatments may induce condensation/repolymerization reactions among lignin fragments, explaining the increased lignin particle size and solubility changes after processing at higher temperatures (i.e., 130 °C).

ChCl–LA-based DES serves a dual role: cleaving the linkages between propylphenol units and selectively extracting lignin fragments from straw, with hydrogen bond interactions explaining the mechanism of DES in removing lignocellulosic biomass.^40^ The hydrogen bond interactions between DES and LNMs allow DES to stretch the hydrophilic bases of LNMs to the surface, forming self-assembled spherical LNM particles, as depicted in Figure 2. After adding deionized water to the LNM mixture, the more substantial hydrogen bonding capability of water molecules can replace that of DES, thus allowing LNMs to be easily separated from DES by deionized water.

The ChCl–LA DES also exhibits significant ability to disperse lignin particles,^41,42^ potentially offering promising applications. The ChCl–LA DES may also play a role in disrupting the electrostatic interactions between lignin particles. Based on SEM images, the size of lignin nanoparticles is estimated using ImageJ software. The values presented in Table 2 are the average of at least 50 particles counted from each lignin sample, further confirming the significant effect of processing temperature on lignin size distribution. In summary, the particle size of lignin samples remains within a narrow range of 400 to 700 nm, with the smallest particle size observed at 120 °C. Limited depolymerization of LNMs occurs at 110 °C, while repolymerization of lignin may occur at 130 °C.

**Table 2:** Average Particle Size of LNMs (120 °C)

	LNM_8h_	LNM_12h_	LNM_16h_
feretX (nm)	655.8	579.5	668.2
feretY (nm)	438.8	407.1	458.3
average (nm)	547.3	493.3	563.25

### 4.3 Physical, Chemical, and Mechanical Characteristics Analysis of the Composites

**Figure 3:**
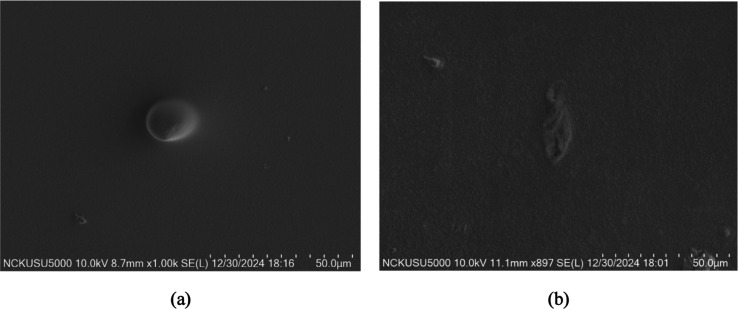
SEM images of PVA/Gly-PEDOT:PSS (a) and PVA/Gly-PEDOT:PSS/CNCs/LNM (b) composite surface.

The SEM images of the composites is shown in Figure 3. The surface of the PVA/Gly-PEDOT:PSS sample (Figure 3a) appears relatively smooth and homogeneous, with minimal surface features, which is typical for a well-mixed polymer matrix without additional fillers. In contrast, the surface of the PVA/Gly-PEDOT:PSS/CNCs/LNM composite (Figure 3b) exhibits increased roughness and noticeable microstructures, likely caused by the inclusion of CNCs and LNMs. These features suggest the presence of CNC and LNM aggregates or interactions that disrupt the uniformity of the polymer matrix.

Similar findings have been reported in previous studies where SEM analysis was performed to investigate the microstructure and dispersion of nanocellulose into PVA biocomposites, demonstrating that as nanocellulose content increases, the surface roughness of the biocomposites also increases due to agglomeration of the nanofibers. The cross-sectional analysis of nanocellulose-reinforced PVA films further revealed random dispersion of nanocellulose without precipitation during the casting process, resulting in a uniform cross section without layer differentiation.^43^

The rougher surface of the composite is indicative of the integration of these nanomaterials, which can enhance properties such as mechanical strength or adhesion. However, the increased roughness may also introduce surface irregularities, emphasizing the need for optimizing the composition of CNCs and LNMs to achieve a balance between improved performance and maintaining surface uniformity. The observed morphological differences confirm the successful incorporation of CNCs and LNMs into the PVA/Gly-PEDOT:PSS matrix, significantly influencing the surface characteristics.

**Figure 4:**
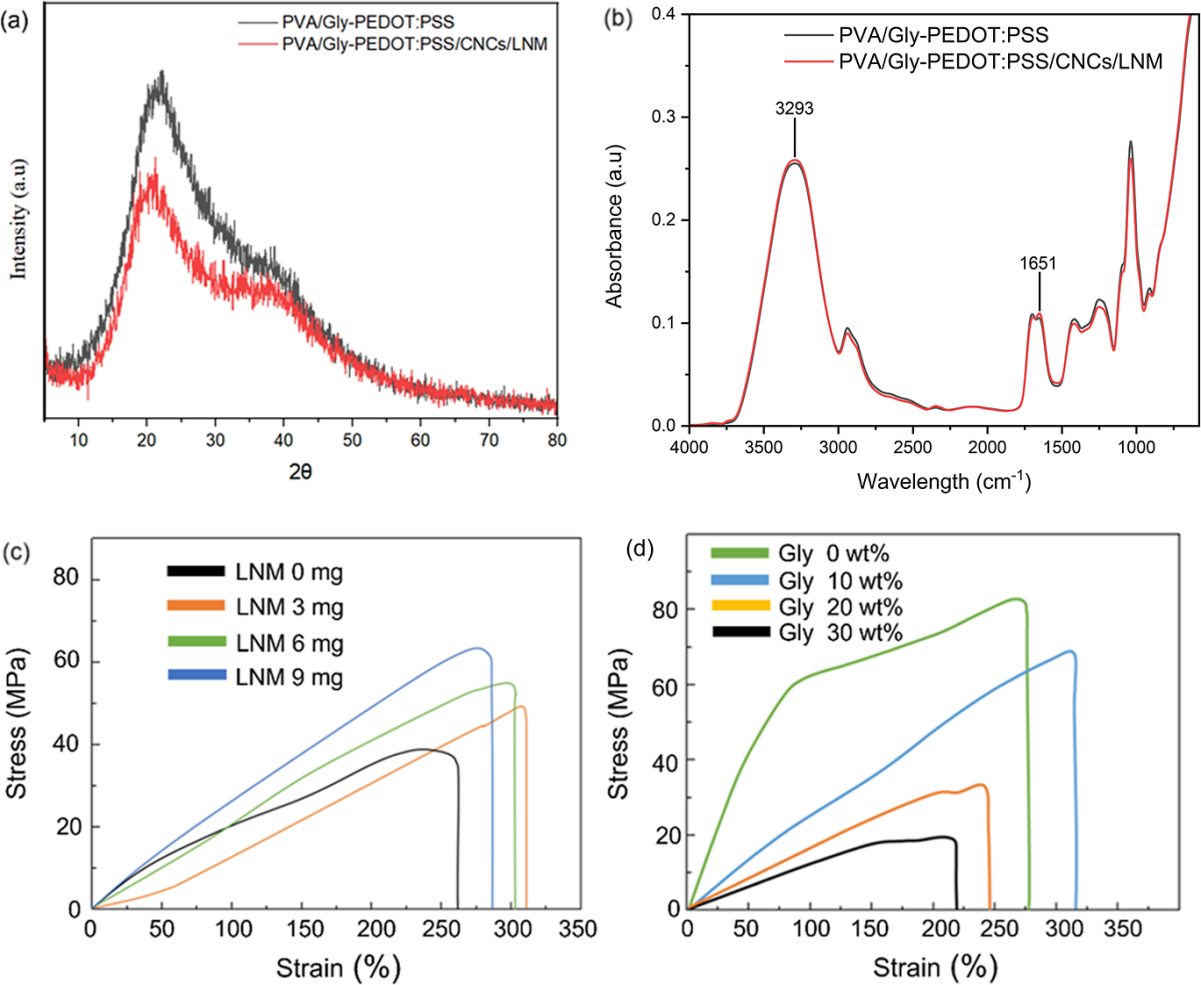
XRD (a) and FTIR spectra (b) of samples with and without CNC-LNM. Tensile test results with various LNM contents (c) and Gly contents (d).

The XRD and FTIR patterns of the PVA/Gly–PEDOT and PVA/Gly–PEDOT/CNCs/LNM composites are shown in Figure 4a,b. The broad halo observed around 2θ ≈ 20° suggests that the materials exhibit amorphous-like characteristics. The addition of CNCs and LNMs results in a reduction in intensity, which is likely caused by a dilution effect. Furthermore, the introduction of CNCs and LNMs disrupts the molecular packing of PVA chains. Lignin’s inherently amorphous structure, combined with strong hydrogen bonding interactions between the carboxyl groups on LNMs and the hydroxy groups on PVA, contributes to the formation of a more disordered matrix.

For FTIR (Figure 4b), the broad peak at 3293 cm^–1^ corresponds to the O–H stretching vibrations, primarily from PVA, glycerol, and any hydroxyl groups present in the added CNCs and LNMs.^43,44^ The absorbance in this region is slightly enhanced for the red spectrum, indicating an increased number or interaction of hydroxyl groups due to the CNCs and LNMs. The peak at 1651 cm^–1^, attributed to the C=O stretching vibrations from the PVA matrix and possibly residual water, shows a slight change in intensity and position, reflecting interactions between the PVA matrix and the CNCs or LNMs.^45,46^

The uniaxial tensile mechanical properties of the composite material are shown in Figure 4b,c. Adding LNM increases the strength and maximum strain rate, as shown in Figure 4b. Increasing the LNM content enhances the sensor’s strength, albeit with a slight reduction in toughness. However, compared to the original material, there is still a significant improvement. Lignin contains a large number of rigid phenolic structures. In the modified LNM, the surface is enriched with a large number of carboxyl groups that form strong hydrogen bonds with PVA. These hydrogen bonds connect the PVA chains and create temporary links that can break and reform. This makes it easier for the PVA chains to move and adjust under stress, improving their flexibility and flow.^47^ At the same time, these bonds help the material stay strong and tough, which is important for sensor applications. In addition, CNC plays a distinct role in improving the mechanical properties of the composite. The structure of CNC acts as a reinforcing filler, enhancing Young’s modulus and tensile strength of the material. The uniform dispersion of CNC within the PVA matrix contributes to stress transfer and provides rigidity without compromising flexibility.

As shown in Figure 4c, Young’s modulus of the PVA/Gly–CNCs/PEDOT:PSS composite material decreases with the increase in glycerol content. Young’s modulus reaches 65.9 MPa at a glycerol content of 10 wt %, with a high stretchability of 320%. The enhanced ductility is due to the abundance of OH bonds in glycerol, improving the internal flowability of the PVA chains making it suitable for use as a strain sensor for human motion detection.

### 4.4 Self-Powered Thermoelectric Properties of Composites

**Figure 5:**
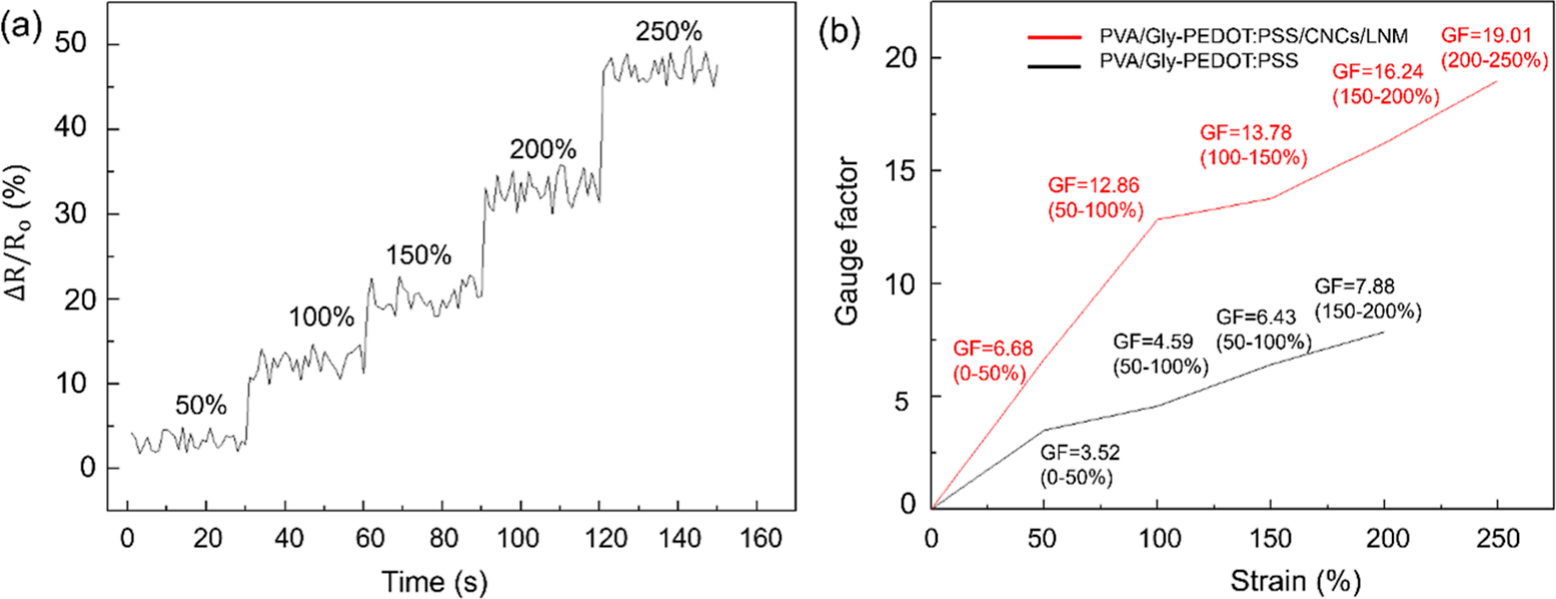
Resistance change rate (a) and gauge factor under various strains (b).

The fabricated sensor demonstrated excellent strain sensitivity and superior stretchability by measuring the relative change in resistance upon strain, as shown in Figure 5a. The relative resistance response and calculated GF values of PVA/Gly–CNCs/LNM/PEDOT:PSS and PVA/Gly–PEDOT:PSS within a 0–250% strain range indicate that the distance between the CNC/LNM/PEDOT conductive fillers expands, leading to an increase in relative resistance with applied strain, thereby generating an instantaneous response of strain to relative resistance values.^48,49^ Moreover, the relative change in resistance occurs exponentially rather than linearly.^50−52^ The GF value of sensors with LNM added is significantly higher than those without LNM, as depicted in Figure 5b. This result is attributed to the higher conductivity of the PVA/Gly–CNCs/LNM/PEDOT: PSS films due to the formation of a percolation network.^53,54^

### 4.5 Analysis of the Self-Powered Thermoelectric Performance

**Figure 6:**
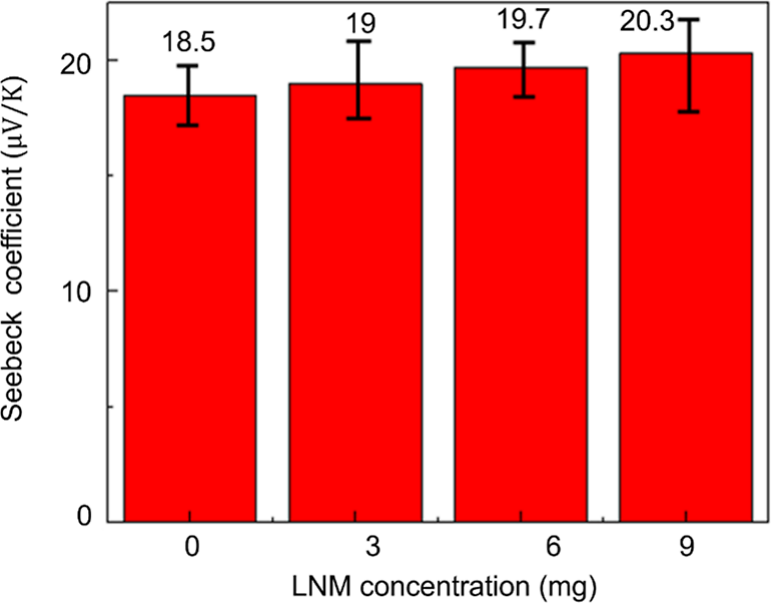
Seebeck coefficients of various LNM contents.

Figure 6 shows the Seebeck coefficients of composite films composed of different amounts of LNMs, with measured values for 0 mg, 3 mg, 6 mg, and 9 mg of LNMs being 18.5, 19, 19.7, and 20.3 μV/K, respectively. In recent work, the Seebeck coefficient of composite films with different CNC fractions was measured, and the addition of CNC did not change the film’s Seebeck coefficient.^55^ In this study, the increase in the Seebeck coefficient with the addition of LNM indicates that LNM plays a key role in enhancing the thermoelectric performance. This enhancement is attributed to the rigid phenolic structures and carboxyl groups on the LNM surface, which improve charge transport and thermal gradient responsiveness within the composite matrix.

**Figure 7:**
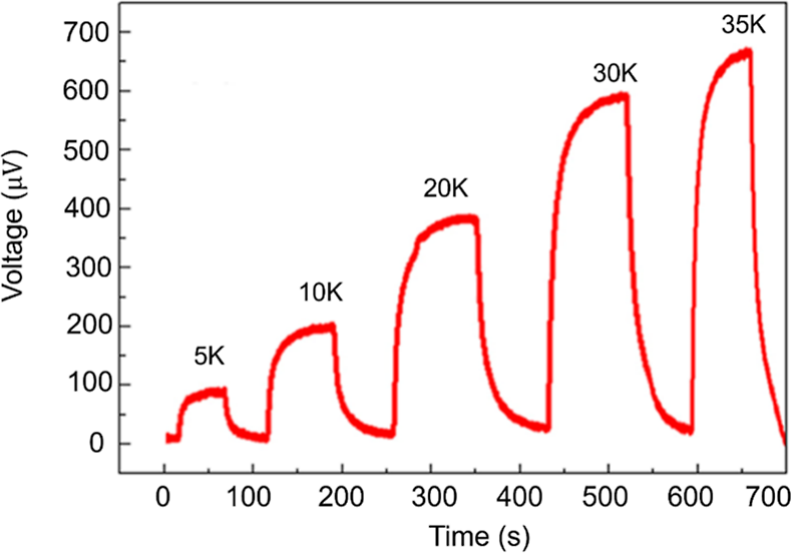
Thermoelectric voltage under various temperature differences.

Conversely, the uniform dispersion of CNC ensures stress transfer and prevents excessive deformation during thermoelectric measurements, which is critical for maintaining consistent performance under strain. Figure 7 displays the voltage response of the sensor under different temperature differences from 5 to 30 K and a self-powered condition at a 30 K temperature difference. The results suggest that CNC’s mechanical reinforcement minimizes deformation under these thermal conditions, ensuring steady voltage output.

**Figure 8:**
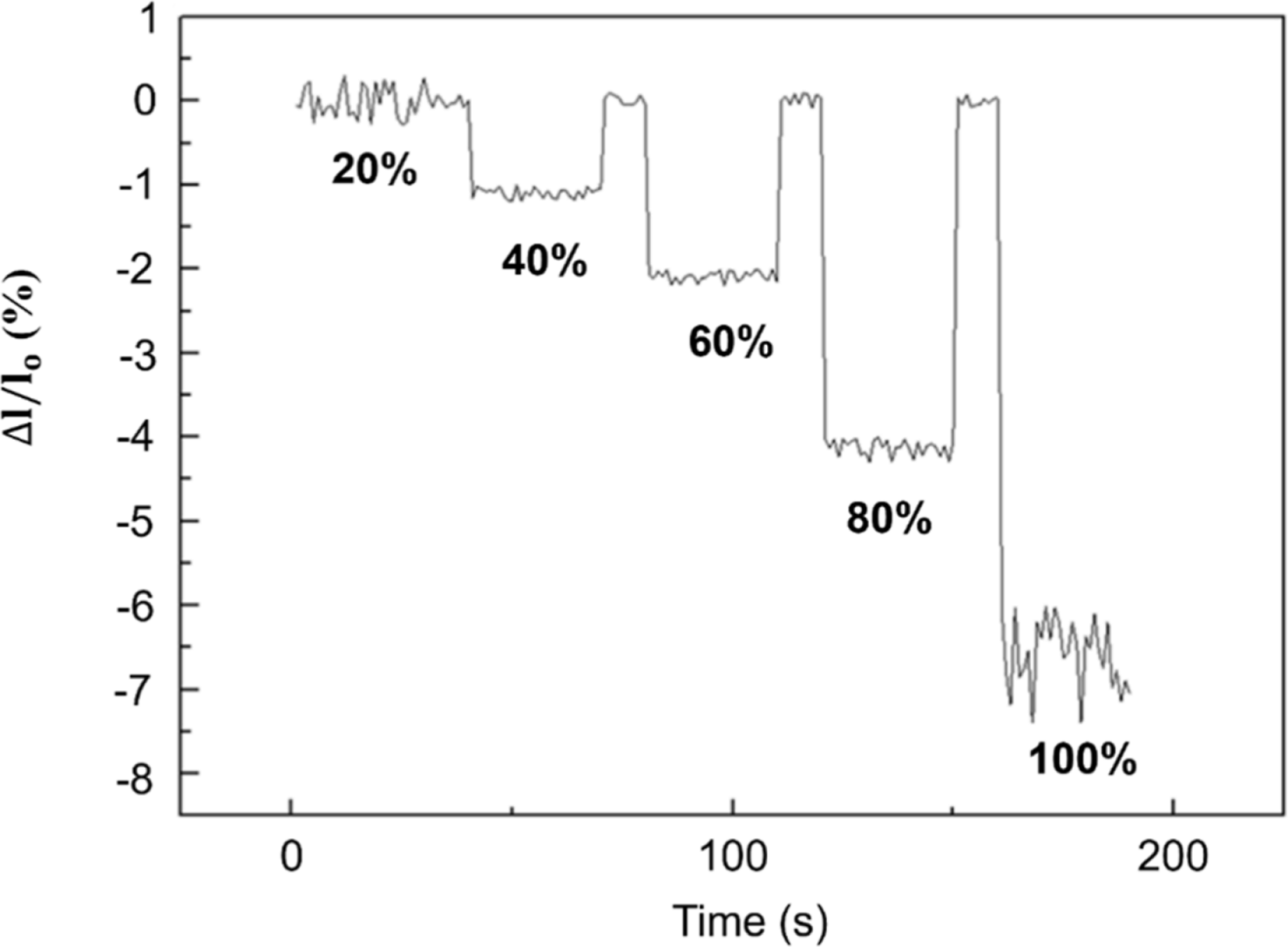
Thermoelectric current change rate under various strains.

Applying 0–100% strain at the device ends significantly changes the current with increasing strain. Finally, at 100% strain, the sensor’s thermoelectric current change amplitude is extremely high, indicating that the self-powered thermoelectric performance fails at 100% strain, as shown in Figure 8.

**Figure 9:**
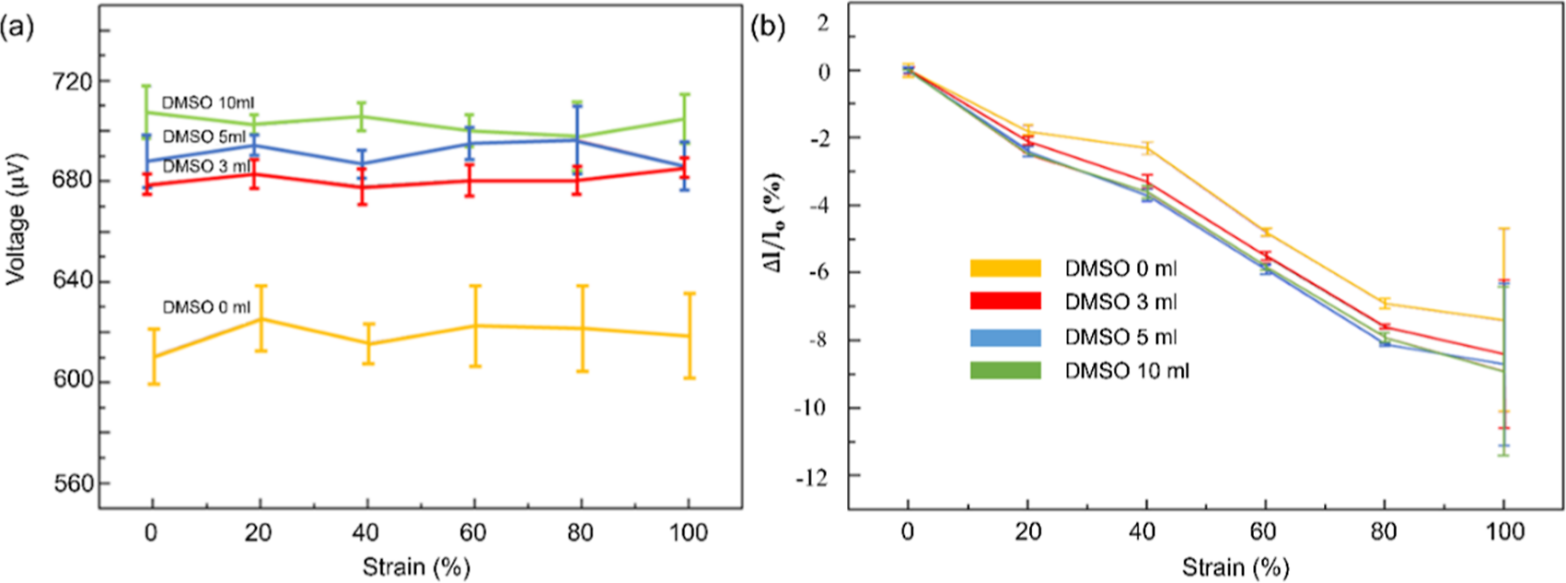
Thermoelectric voltage (a) and current of various DMSO contents (b) under various strains.

The morphological changes in the PEDOT: PSS layer with added DMSO can be attributed to the bridging phenomenon among PEDOT chains during the crack propagation. By analyzing the thermoelectric voltage change and thermoelectric current change with three different DMSO contents, we can visualize that the thermoelectric effect of PVA/Gly–CNCs/LNM/PEDOT: PSS significantly improves after adding DMSO, as shown in Figure 9a. A higher DMSO content increases self-powered thermoelectric voltage under a 30 K temperature difference, as shown in Figure 9b. The strain relative to the change in self-powered thermoelectric current also increases. This is due to the bridging mechanism between PEDOT chains and the improvement in cohesion and conductivity after an appropriate amount of DMSO doping.^56^

### 4.6 Fatigue Testing and Detection of Human Motion Behavior

**Figure 10:**
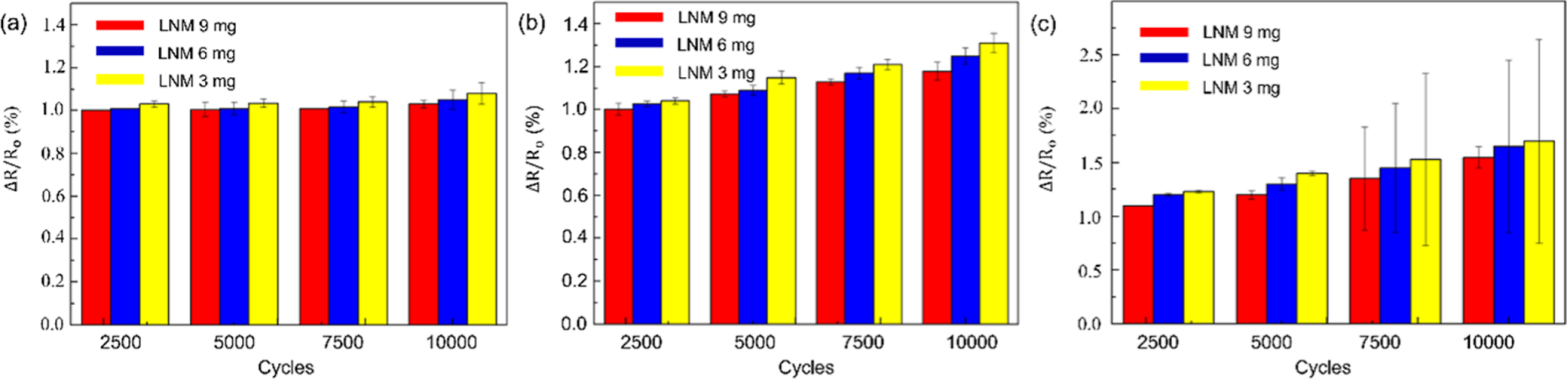
Resistance rate under 0–25% (a), 0–50% (b), and 0–100% (c) strain cyclic stretching test.

**Figure 11:**
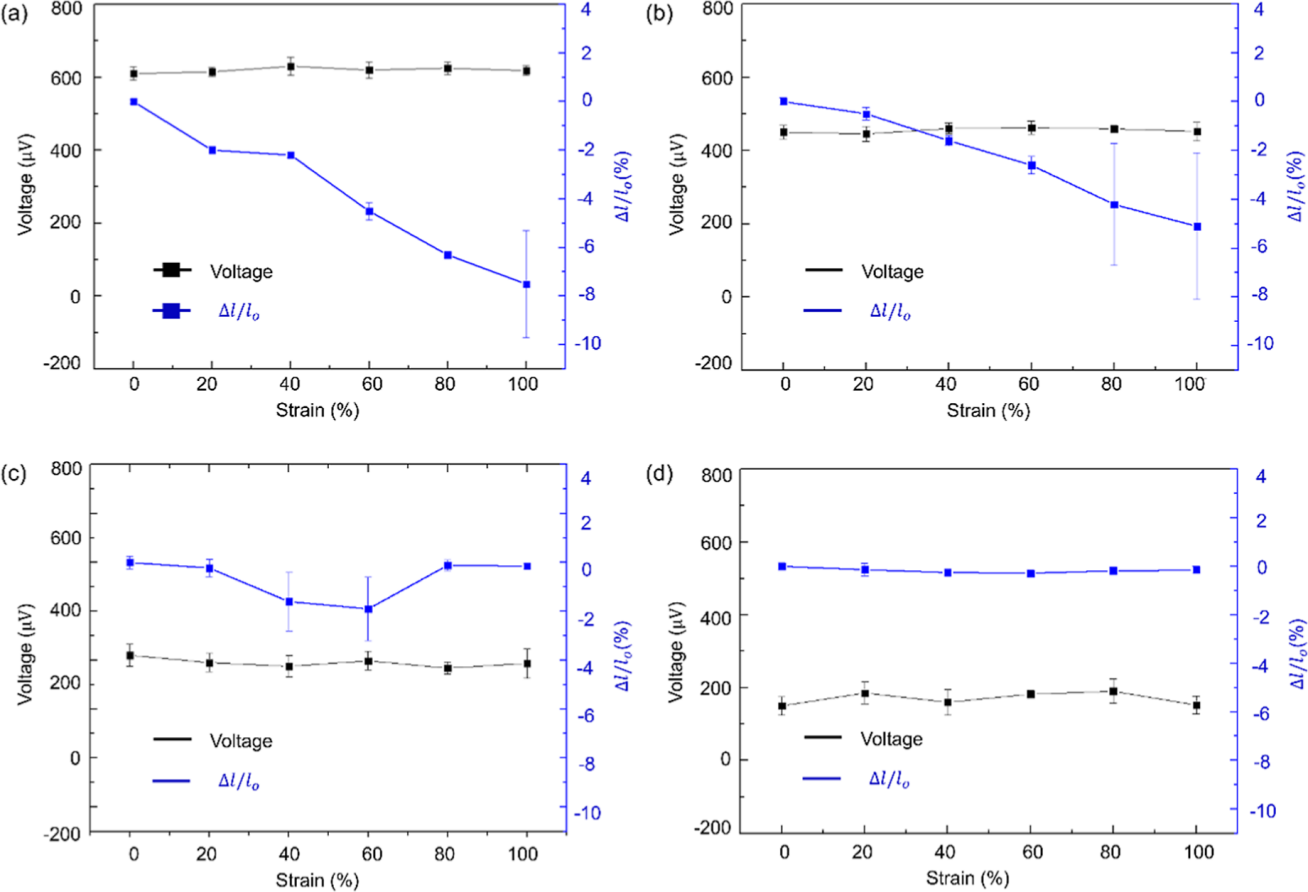
Testing the self-powered thermoelectric properties with 9 mg of LNM at 100% strain after 2500 (a), 5000 (b), 7500 (c), and 10,000 (d) reciprocations.

The mechanical durability is represented in Figure 10a, demonstrating that even after 10,000 cycles of reciprocating stretching from 0–25% strain, there is no significant change in the sensor’s resistance rate. This illustrates our sensor’s good flexibility, and it is observed that the sensor with 9 mg of LNM content shows a minor resistance rate change after 10,000 cycles of reciprocating stretch tests compared to the other two groups. This indicates that adding LNM can enhance the sensor’s flexibility, stemming from the excellent compatibility of LNM with the PVA substrate and the multihydrogen bond structure of LNM, improving the flowability of PVA molecular chains and making the sensor more flexible. Figure 10b,c displays the relative resistance changes for the 0–50% and 0–100% strain range after 10,000 cycles of reciprocating stretch tests, respectively. The sensors in this study show only minor changes in resistance rate under 10,000 cycles of stretching tests from 0–50% strain. Figure 11 illustrates the self-powered thermoelectric properties of the 9 mg LNM sensor under 5000 cycles of stretching tests at 100% strain in a 30 K self-powered condition, showing 0–100% stable strain response, indicating the sensor has good repeatability and durability as a strain sensor.

**Figure 12:**
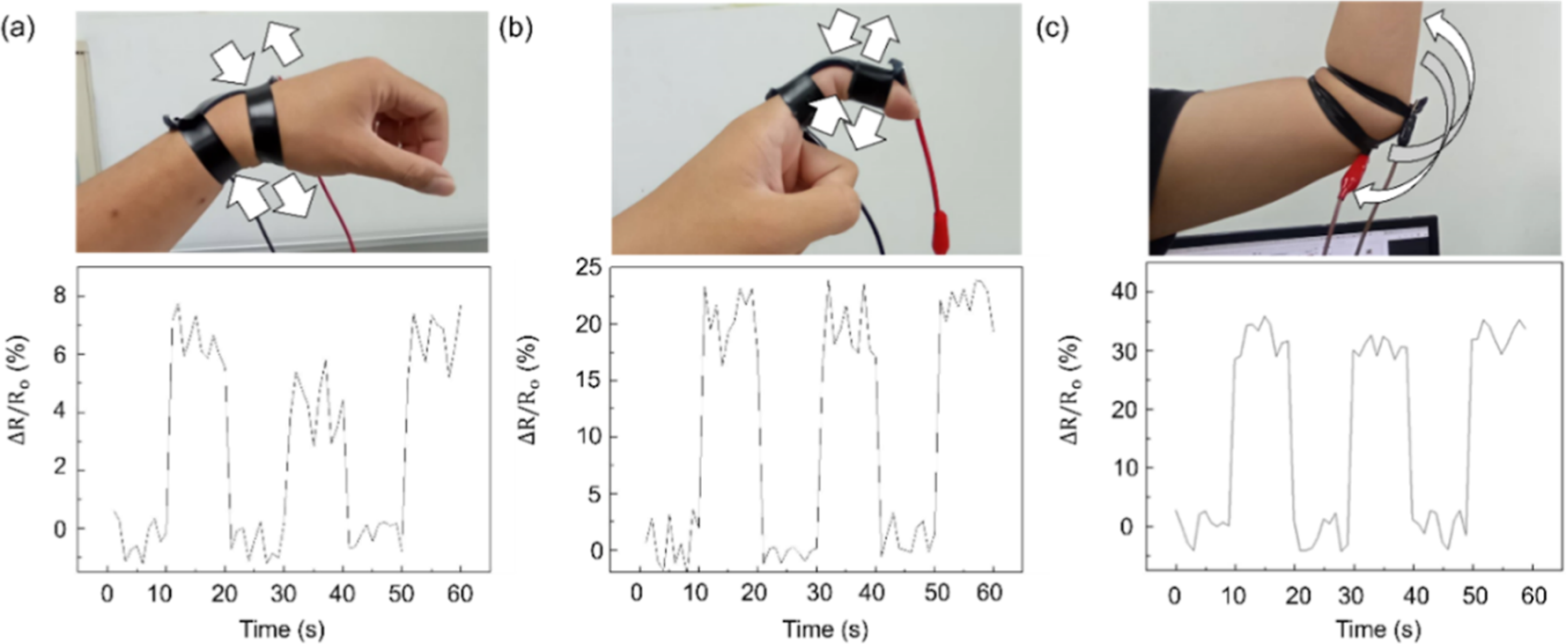
Resistance change rate for various strains on the wrist (a), finger (b), and elbow (c).

PVA/Gly–CNCs/LNM/PEDOT: PSS sensors detected joint movements, as presented in Figure 12. The sensor’s average response time was approximately 0.84 s, with recovery times averaging 1.12 s across all tested configurations. When the finger bends at a certain angle, the sensor’s resistance value gradually increases; when the finger maintains a certain angle, the resistance value remains unchanged. Moreover, due to the sensor’s high sensitivity and low modulus, it can also monitor subtle human movements, which is beneficial for detecting minor skin deformations.

## 5 Conclusion

In summary, the PVA/Gly–CNCs/LNM/PEDOT composite demonstrates significant advancements in both mechanical and strain-sensitive properties, resulting in an efficient, self-powered strain sensor ideal for wearable technology. By refining the preparation process of LNMs through a 12 h autoclave-based approach, the composite material’s production becomes faster and more efficient than traditional 48 h methods. The addition of LNM enriches the PVA matrix with carboxyl groups, forming strong hydrogen bonds that improve the sensor’s toughness, rigidity, and flowability. At a glycerol content of 10 wt %, the composite achieves Young’s modulus of 65.9 MPa and stretchability up to 320%, which is sufficient for detecting human movement, although slightly below the initial target of 500%.

Furthermore, CNCs and LNMs enhance the gauge factor, which increases the sensor’s strain sensitivity, making it highly responsive to subtle movements. The high aspect ratio of CNC fillers enables the formation of an efficient electrical network within the PVA matrix, reducing the percolation threshold and enhancing the gauge factor to 19, close to the target of 20. This sensor also generates a thermoelectric voltage across a temperature differential of 5–35 K, with stable responses across a constant 30 K difference under strains from 1% to 100%, highlighting the role of LNM’s phenolic structures in improving electrical flowability and dispersion of PEDOT.

After 5000 cycles of 100% strain testing under a self-powered condition, the sensor displays excellent durability, maintaining consistent strain responses and demonstrating its potential for repeated use. The PVA/Gly–CNCs/LNM/PEDOT composite material has been successfully applied to detect movements of human fingers and wrists when adhered to the skin, showcasing its potential in wearable and motion detection applications.

Additionally, the composite material can be adapted to various manufacturing processes, such as 3D printing and electrospinning, offering versatility for scalable production. This environmentally friendly, low-cost sensor design strategy provides an effective path for developing highly stretchable and conductive composites. Future applications could include not only human motion detection but also robotic electronic skin, setting a foundation for future developments in artificial sensory systems for human-machine interfaces.
